# Role of the Media in Health-Related Awareness Campaigns on Perception of COVID-19: A Pre-post Study in the General Population of Pakistan

**DOI:** 10.3389/fpubh.2021.779090

**Published:** 2021-11-12

**Authors:** Atta Ur Rehman, Rubeena Zakar, Muhammad Zakria Zakar, Ume Hani, Kamil J. Wrona, Florian Fischer

**Affiliations:** ^1^Institute of Social and Cultural Studies, University of the Punjab, Lahore, Pakistan; ^2^University of Okara, Punjab, Pakistan; ^3^Holy Family Hospital, Rawalpindi Medical University, Rawalpindi, Pakistan; ^4^School of Public Health, Bielefeld University, Bielefeld, Germany; ^5^Institute of Public Health, Charité—Universitätsmedizin Berlin, Berlin, Germany; ^6^Institute of Gerontological Health Services and Nursing Research, Ravensburg-Weingarten University of Applied Sciences, Weingarten, Germany

**Keywords:** health communication, health education, community education, electronic media, SARS-CoV-2

## Abstract

Medical preparedness and community education are the most valuable preventive tools for combatting the COVID-19 pandemic. This study aims to assess the role of media public health awareness campaigns on the knowledge of the general population about COVID-19 in Rawalpindi, Pakistan. A quantitative study using a pre-post design among 384 respondents was conducted. A structured questionnaire was administered to the participants twice: The first response (*t*_1_) from participants was filled in during the 1st week in February 2020 before any confirmed cases were reported in the country, and the second response (*t*_2_) was completed 1 month after the first case detection in Pakistan (March 2020). Media health awareness campaigns were launched just after the detection of the first case in Pakistan. Exposure to the media and knowledge relating to COVID-19 increased over time. Whereas, only a quarter of respondents judged the isolation of suspected cases in quarantine to be important to prevent the spread of infection in society at *t*_1_, more than half did so at *t*_2_. Socio-demographic characteristics were not significantly associated with knowledge (gains). However, more frequent use of electronic media is associated with greater knowledge gains from *t*_1_ to *t*_2_. The findings of this study provide evidence that awareness and knowledge related to COVID-19 symptoms and preventive measures increased significantly over time. The increased frequency of following the media indicates that health awareness campaigns are important for enhancing the knowledge of the general public regarding COVID-19.

## Introduction

On 30 January 2020, the World Health Organization (1) declared the Novel Coronavirus Disease 2019 (COVID-19) outbreak to be a global public health emergency. Coronaviruses are a microbial source of infections in individuals, with a spectrum of activity associated with the common cold, Severe Acute Respiratory Syndrome (SARS), and Middle East Respiratory Syndrome (MERS) (2). The outbreak of a pneumonia of unknown cause was observed in Wuhan, China, in December 2019 where a novel coronavirus (SARS-CoV-2) was isolated from patients in January 2020 (3). This outbreak—and the associated strict isolation—attracted global attention due to health communication by the media networks. The movement of people along the road passage from Iran and air travel from other countries brought the virus to Pakistan, which confirmed its first case on 26 February 2020 (4). The number of confirmed COVID-19 cases in Pakistan was 523,011 on 19 January 2021, with 11,055 confirmed deaths (5).

Medical preparedness and community education are the most valuable preventive tools for combatting the pandemic (6, 7). The media has already been serving as an important source of health education and promotion in our societies for decades (8). Health-related communication campaigns in the media have aimed to change the health behavior of the population by creating awareness and promoting prevention, such as hand hygiene practices and immunization coverage (8–10). Health campaigns are categorized into typical and digital technology use campaigns. Typical communication involves the use of different media channels (e.g., print media or electronic media), whereas digital technology communication may involve the use of mobile phones and internet web search engines (8). Health communication plays a vital role in behavioral changes (11) and may, finally, result in modifications in the awareness, attitudes, and practices of the targeted audience for improving health.

After the report of first case in Pakistan, the government of Pakistan, in collaboration with the WHO, established isolation units in leading hospitals, set up screening facilities at border entry points, seaports and airports, facilitated quarantine areas at selected places, provided personal protective equipment for healthcare professionals, and enforced lockdown in cities to break the chain of infection (12).

In Pakistan, the media started to report about the epidemic when it first appeared in Wuhan, but its reporting increased drastically, with more focus on preventive measures, after the first reported case on 26 February 2020 (4). Within the national action plan to combat COVID-19, the Pakistani government developed a national risk communication and community engagement strategy. This strategy included dissemination of COVID-19 related information to the general public through media. The national website on COVID-19 provides information on awareness along with morbidly, mortality, and testing statistics of the country on daily basis. Awareness campaigns launched nationally were categorized into typical and digital technology use campaigns: Typical communication involved the use of newspapers, television, and radio, whereas digital technology communication covered the use of mobile phone messages and internet web search engines. Consultation on COVID-19 was provided by a national helpline and telemedicine departments of the medical universities (5, 13).

The COVID-19 related awareness campaign in Pakistan emphasized on the symptoms of the disease, preventive measures, and the importance of physical distancing (5, 13). Symptoms of a coronavirus disease may appear within 2–14 days after viral exposure. The initial symptoms may include fever, cough, headache, sore throat, shortness of breath, and rapid heartbeat, with complications of pneumonia and organ failure. Treatment options are only supportive because no targeted anti-viral therapeutics are available at present (14). The elderly, persons with diabetes, and immune-compromised people are the most vulnerable groups when infected with COVID-19 (15). The general public has lack of access to vaccination against COVID-19 as it is not yet available in Pakistan (16). Protective measures adopted by the general population should include frequent handwashing, use of hand sanitizers, wearing face mask, avoiding close contact with sick people, and physical distancing practices (17).

Correct perception of population is essential to ensure good preventive practices for control of corona pandemic (18). Population based media exposures reported positive or prevent negative changes in behaviors (8). Health-related awareness campaigns regarding polio vaccination, family planning, and acquired immunodeficiency syndrome prevention were successful in Pakistan in creating awareness among masses and encouraging people to use healthy behaviors (19). Given this backdrop, there is an urgent need to investigate the role of media as an awareness creator regarding COVID-19 signs, symptoms and protective measures to prevent its transmission.

Our study is based on the Knowledge Gap Hypothesis. This hypothesis proposes that knowledge is disseminated in the society on the basis of socio-economic indicators. The philosophical stance of the knowledge gap hypothesis described that people with better financial status may assimilate media information more rapidly than lower financial status (20). The socio-demographics association with awareness was analyzed in this study. The objectives of the study were to assess the role of media health-awareness campaigns on the general population's perception and knowledge of COVID-19 in Rawalpindi, Pakistan.

## Methods

### Study Design

A quantitative research method based on a pre-post study design was used to collect data from the general population in Rawalpindi, which is the fourth most populous city in Pakistan and located in Punjab province (21). Rawalpindi is adjacent to Pakistan's capital of Islamabad and is an important administrative, commercial and industrial hub. In addition to urban settlements, it comprised of numerous suburban housing developments that provide residence to workers in Islamabad. Being close to the country's capital, Rawalpindi has active media networks and a large number of cable TV service providers.

### Data Collection

Data was collected during the first wave of the COVID-19 pandemic. At the time of the first wave, Rawalpindi was among the top three cities with highest numbers of COVID-19 cases in Punjab (22). At that time, the country was experiencing a complete lockdown except for healthcare facilities and pharmacies which were allowed to practice. This was the reason that the present study was conducted in a community pharmacy of Rawalpindi. Secondly, community pharmacies were the places that were frequently visited by the general public for prescription refilling for relatives, health accessories, and cosmeceutical purchases. Thirdly, the visitors' record including their contact information was well maintained at the pharmacy, which was essential for the post-phase of the study. Nonetheless, there were some limitations in selecting the pharmacy as study site. This particular location may result in an exclusion of certain groups of people (e.g., people with good health or people who cannot afford to go to a pharmacy). However, this was the most suitable location at the time of the pandemic and its associated lockdown.

The respondents were regular clients of the community pharmacy in Rawalpindi, who visited every month. The adult population using the media as a source of health awareness, not currently labeled as patients by any prescriber, and being willing to participate were included in the study.

Face-to-face interviews were conducted by the first author in the sitting area of the pharmacy (which was arranged on the request of researchers in a corner of the pharmacy). The average time for each interview was 12 minutes. The response rate at *t*_1_ was 100%. Paper-and-pencil questionnaires were filled by the interviewer. After the interview, the respondents were informed about the second phase (*t*_2_) of data collection and their willingness and contact information were asked for contacting them again. The respondents were informed about the timing of the second phase through phone or SMS message. Most of the interviews were conducted at the pharmacy at respondents' convenience whenever they visited the pharmacies for prescription refilling for relatives, buying health accessories, and cosmeceutical purchases. However, seven of the respondents could not visit the pharmacies and gave their responses on phone. The non-response rate was 3% in the second phase and 2% of questionnaires were incomplete. For that reason, the data of 384 respondents were included in the final analysis.

### Sample Size and Sampling Technique

The sample size of 384 was calculated on the hypothesis that knowledge prevalence related to covid-19 sigs, symptoms and preventive measure's (*P*) would be 50%, with an allowable error (*d*) of 5% and a confidence level of 95% (*z* = 1.96). A non-response rate of 5% was added and the final sample size was 403. The technique used for the data collection was systematic random sampling, because the public was rationally similar. Each wave of the study needed to be completed within 1 week. As the average population visiting the pharmacy every week was *n* = 1,920, the sampling interval was *k* = 5. The first respondent was chosen in February by using a software method for simple random sampling. The first respondent selected was visitor number 3. Then, by the addition of participants at the regular interval (*k* = 5), the sample size was completed. The same participants completed the second response.

### Items of Interest

A self-designed structured questionnaire (Supplementary Appendix 1) was used as toll of data collection. The tool consisted of three sections; the first section comprised of information on the socio-demographics characteristics of the respondents including age, gender, marital status, place of residence, level of education, and family monthly income. The second section was related to the history of the frequency of using different types of media for information seeking about COVID-19. The respondents were asked about the frequency of use of different types of media such as social/digital (Facebook, WhatsApp, twitter, internet, websites of public bodies, health portals), electronic news portals (television), and print media (newspaper, magazines, brochures) for public health awareness related to COVID-19. The third part of the questionnaire was related to knowledge regarding COVID-19 symptoms, complications, and preventive measures to be adopted regarding COVID-19, and the effect of lockdowns on social isolation. The respondents were asked to answer whether several statements were correct (“Yes”, “No”, or “Don't know”).

Correct statements were valued with one point each and summed (without weighting) in three subscales related to knowledge (general, symptoms, and preventive measures) and an overall total scale (ranging from 0 = “No knowledge” to 19 = “Full knowledge”). The subscales included a five-item subscale related to general awareness (i.e., coronavirus is contagious, spread through droplets, spread through coughing and sneezing by an infected person, coronavirus treatment is only supportive, and no vaccine is available), a six-item subscale on symptoms and complications (i.e., fever, cough, body aches, shortness of breath, pneumonia, organ failure), and an eight-item subscale on preventive measures (frequent handwashing with soap and water, following cough and sneeze etiquette, avoiding social contact with people, use of face masks, use of hand sanitizers, isolation of suspected cases, their perceptions regarding lockdown as preventive measures, for example, lockdown helped people in following physical distancing and lockdown helped in protecting people from the spread of infection). The questionnaire's construct validity and reliability were evaluated by factor analysis and Cronbach's alpha, respectively. The Kaiser-Meyer-Okin measure was 0.89 with significant Bartlett's test. Three components were extracted to measure the underline construct. The reliability Cronbach alpha value was 0.899 for the subscale of general awareness, 0.922 for the subscale of symptoms and complications, and 0.873 for the preventive measure subscale.

### Media Awareness Intervention

This study investigates the role of the media in shaping the perceptions of the general population visiting a community pharmacy in Rawalpindi, Pakistan, toward COVID-19. The first response from participants was filled out during the 1st week of February 2020 before any confirmed cases were reported in Pakistan (*t*_1_). Media awareness and prevention campaigns for COVID-19 started just after the detection of the first case on 26 February 2020, and reached a peak in March 2020 in Pakistan. Lockdown in the country also created curiosity related to COVID-19 in the general public (23, 24). The national disaster management authority, Pakistan's telecommunications authority, as well as electronic and print media were continuously providing awareness alerts and preventive communications. The health education and prevention interventions by the media comprised of comprehensive education on coronavirus awareness (e.g., current spread, transmission routes, or symptoms of COVID-19), along with the screening and preventive measures (individual measures to protect against infections, hygiene regulation, dealing with mental stress during the COVID-19 lockdown) that needed to be adopted to stay healthy and safe from COVID-19 (13). During the last week of March, the second response was collected from each participant (*t*_2_), giving an 8-week interval between the two surveys.

### Data Analysis

The data was analyzed using SPSS version 21. We applied descriptive and inferential statistical methods. Frequencies and percentages were computed for summary statistics. We used correlation tests for the association between the different media types. The research aims to describe the potential change in population perceptions regarding COVID-19, following media campaign exposure during February and March 2020. Statistical tests such as the paired *t*-test and chi-square test were used to assess changes in the population's perceptions during subsequent months.

The factors associated with knowledge were assessed using three linear regression models. The dependent variables were the overall scores for knowledge related to COVID-19 at *t*_1_ and *t*_2_, and for the knowledge gains over time (between *t*_1_ and *t*_2_). Independent variables were the variables related to media use and socio-demographic characteristics, such as those described in Table 1, except for education, which was categorized as a binary variable (“12 years or fewer” vs. “13 years or more”) to allow for large enough sub-groups in the regression models. The R2 was calculated as the coefficient of determination.

**Table 1:** Sociodemographic characteristics of respondents (*n* = 384).

**Sociodemographic characteristics**	**n (%)**
**Age (in years)**	
16–30	59 (15.4)
31–45	62 (16.1)
46–60	178 (46.4)
61–75	85 (22.1)
**Gender**	
Male	308 (80.2)
Female	76 (19.8)
**Marital status**	
Currently married	263 (68.5)
Currently not married	121 (31.5)
**Place of residence**	
Urban	200 (52.2)
Rural	184 (47.8)
**Level of education**	
<11 years of education	96 (25.0)
11-12 years of education	145 (37.8)
13–14 years of education	96 (25.0)
15–16 years of education	28 (7.3)
>16 years of education	19 (4.9)
Family monthly income (in Pakistani rupees^*^)	
<25,000	104 (27.1)
25,000–50,000	126 (32.9)
50,001–75,000	112 (29.1)
75,001–100,000	34 (8.9)
>100,000	8 (2.0)

* *1 US Dollar = 166.65 Pakistani rupees*.

### Ethical Considerations

The study protocols were reviewed and approved by the Institutional Ethical Review Board, University of the Punjab (143/IERB/PU). The investigation's objectives were clearly explained to participants before the questionnaires were administered, and written informed consent was obtained. Respondents were informed about the ethics and their right of voluntary participation. The respondents were guaranteed confidentiality and anonymity of their responses in the publication.

## Results

### Sociodemographic Characteristics

The majority of participants were middle-aged. About 80.2% of respondents were male and 68.5% were married. A majority, 62.8%, of the participants did not have a university degree and 60.0% were earning <50,000 rupees per month. Almost equal proportions were from rural and urban areas (Table 1).

### Use of Various Media Channels

The research investigation involves filling out questionnaires, both before (*t*_1_) and after (*t*_2_) the first reported case of COVID-19 in Pakistan. The media were considered to be an information provider and awareness creator. People use different types of media—either exclusively or in combination—to acquire information. Different types of media correlated at a low or moderate level for each instance of data collection. However, there was a very high correlation for each type of media when comparing *t*_1_ and *t*_2_. The daily users of social media increased from 46.1 to 54.7% from *t*_1_ to *t*_2_. The proportion of weekly users of social/digital media stayed almost the same. Electronic media (news portals) were the most widely used among participants (62.5% at *t*_1_ and 71.7% at *t*_2_). The use of newspapers and magazines decreased significantly, as 64.3% of respondents were not using them in March compared to 45.1% in February (Table 2).

**Table 2:** Frequency of media use before (*t*_1_) and after (*t*_2_) the first reported case of COVID-19 (*n* = 384).

**Type of media**	**Media use at t**_**1 **_			**Media use at t**_**2**_		
	**n (%)**			**n (%)**		
	**Daily**	**Weekly**	**Not follow**	**Daily**	**Weekly**	**Not follow**
Social media (e.g., Facebook, WhatsApp)	177 (46.1)	99 (25.8)	108 (28.1)	210 (54.7)	95 (24.7)	79 (20.6)
Electronic media (e.g., television)	240 (62.5)	72 (18.8)	72 (18.8)	273 (71.7)	59 (15.4)	52 (13.5)
Print media (e.g., newspaper, magazine)	150 (39.1)	61 (15.9)	173 (45.1)	97 (25.3)	40 (10.4)	247 (64.3)

### Awareness and Knowledge Related to COVID-19

The first response in February depicts an overall low level of knowledge regarding COVID-19 among participants. At *t*_1_, 37.5% of respondents knew that the coronavirus is a contagious viral disease, whereas 51.8% were aware of the transmittable nature of coronavirus at *t*_2_. The droplet route of coronavirus transmission was known to 29.2% (*t*_1_) and 42.4% (*t*_2_) of the sampled population. That coughing and sneezing of viral material spreads the infection to healthy people was correctly identified by 36.7% of the respondents initially and that knowledge level had increased to 63.3% in the second response. At *t*_1_, 22.7% of participants knew that coronavirus treatment is only supportive, while 51.6% confirmed this statement at *t*_2_. In February, 15.4% erroneously judged the statement that a vaccine is available to be correct, whereas only 0.5% did so in March (Table 3).

**Table 3:** Correct knowledge related to COVID-19 in February (*t*_1_) and March (*t*_2_) 2020 (*n* = 384).

**Variables**	**t_**1**_**	**t_**2**_**
	**n (%)**	**n (%)**
**Coronavirus general awareness**		
Coronavirus is a contagious viral disease	144 (37.5)	199 (51.8)
Coronavirus spreads via droplet infection	112 (29.2)	163 (42.4)
Coronavirus spreads through coughing and sneezing of the infected person	141 (36.7)	243 (63.3)
Coronavirus treatment is only supportive	87 (22.7)	198 (51.6)
Coronavirus vaccine is available (*wrong statement*)	325 (84.6)	382 (99.5)
**Knowledge regarding symptoms of COVID-19**		
Fever	105 (27.3)	263 (68.5)
Cough	81 (21.2)	249 (64.8)
Body aches	133 (34.6)	262 (68.2)
Shortness of breath	105 (27.3)	259 (67.4)
**Complications of COVID-19**		
Pneumonia	76 (19.8)	94 (24.5)
Organ failure	85 (22.1)	126 (32.8)
**Preventive measures to be adopted for COVID-19**		
Frequent handwashing with soap for 20 s	123 (32.0)	328 (85.4)
Following cough and sneeze etiquette	158 (41.4)	216 (56.3)
Avoid social contact with sick people	109 (28.4)	213 (55.5)
Use of face mask	130 (33.9)	203 (52.9)
Use of sanitizer	139 (36.2)	253 (65.9)
Isolation of suspected cases	109 (28.4)	205 (53.4)
**Lockdown effect in countries during COVID-19**		
Lockdown helped people in following social distancing	106 (27.6)	225 (58.6)
Lockdown helped in protecting people from the spread of infection	93 (24.2)	211 (54.9)

Awareness regarding the symptoms of COVID-19 indicated a noteworthy increase in knowledge among participants. At *t*_1_, about one third provided correct responses to all the different kinds of symptoms, whereas this was about two thirds at *t*_2_. The general public's correct response rate related to complications of COVID-19 was much lower. Fewer than 23% in February and fewer than 33% in March identified pneumonia and organ failure as complications of COVID-19 (Table 3).

The results of the questions about preventive measures to be adopted indicated that 32% of respondents were conscious of frequent handwashing in February. This response had increased significantly to 85.4% in March. For all other preventive measures, the correct responses increased as well, but at a lower level, from about one third correct answers to slightly more than half. Whereas, only a quarter of respondents judged the isolation of suspected cases in quarantine to be important for preventing the spread of infection in society at *t*_1_, more than half did so at *t*_2_. The same increase was visible in relation to the statement that a lockdown helps in following social distancing (Table 3).

### Knowledge Gains Over Time and Associated Factors

The changes within three subscales related to COVID-19 awareness (general, symptoms, and preventive measures), as well as total awareness as the combination of all three subscales, are presented in terms of mean differences in Table 4. For all scales, knowledge increased significantly over time.

**Table 4:** Knowledge related to COVID-19 in February (*t*_1_) compared to March (*t*_2_) 2020 (*n* = 384).

**Variables**	**Time**	**Mean**	**SD**	**Mean difference**	**p-value**
Coronavirus general awareness (5 items)	*t*_1_	2.11	1.43	0.97	<0.001
	*t*_2_	3.08	1.49		
Symptoms of COVID-19 (6 items)	*t*_1_	1.52	2.20	1.74	<0.001
	*t*_2_	3.26	1.49		
Preventive measures to be adopted (8 items)	*t*_1_	2.53	2.69	2.30	<0.001
	*t*_2_	4.83	1.71		
Total (19 items)	*t*_1_	6.16	5.80	5.02	<0.001
	*t*_2_	11.18	3.71		

Using three linear regression models, we analyzed the factors associated with knowledge (all knowledge items combined in one score) related to COVID-19 at *t*_1_, at *t*_2_, and those factors associated with knowledge gains over time (between *t*_1_ and *t*_2_). Socio-demographic characteristics are not significantly associated with knowledge, except for an inverse relationship with income at *t*_2_. Although not significant, people of younger age, female, and living in urban areas had a greater likelihood of better knowledge related to COVID-19 at both *t*_1_ and *t*_2_. Nevertheless, knowledge gains were higher within those groups with lower knowledge levels at *t*_1_. Respondents with a higher educational level had greater knowledge at *t*_1_ and *t*_2_, and also demonstrated greater knowledge gains.

More frequent use of social media and electronic media was associated with lower levels of knowledge in both surveys. Although the use of electronic media is significantly inversely related to knowledge at both *t*_1_ and *t*_2_, this does not hold for the changes in knowledge over time: more frequent use of electronic media is associated with higher knowledge gains from *t*_1_ to *t*_2_ (*B* = 0.522, *p* = 0.018). The variance explained by the variables included in the models is <5% for all three models (Table 5).

**Table 5:** Factors associated with knowledge (gains) related to COVID-19 (*n* = 384).

	**t**_**1**_			**t**_**2**_			**Knowledge gain (t**_**1**_ **to t**_**2**_**)**		
	**B**	**T**	**p-value**	**B**	**T**	**p-value**	**B**	**T**	**p-value**
Age	−0.327	−1.056	0.292	−0.108	−0.553	0.581	0.164	1.016	0.310
Gender	1.021	1.365	0.173	0.639	1.351	0.178	−0.482	−1.234	0.218
Residence	−0.842	−1.413	0.158	−0.722	−1.905	0.058	0.067	0.214	0.830
Education	0.263	0.375	0.708	0.335	0.756	0.450	0.032	0.088	0.930
Income	−0.186	−0.570	0.569	−0.465	−2.210	0.028	−0.293	−1.687	0.093
Social media	−0.305	−0.869	0.385	−0.037	−0.158	0.875	0.042	0.214	0.831
Electronic media	−0.961	−2.462	0.014	−0.575	−2.156	0.032	0.522	2.373	0.018
Print media	0.340	1.025	0.306	0.013	0.058	0.954	−0.156	−0.861	0.390
*Constant*	9.486	4.122	<0.001	13.915	9.184	<0.001	4.474	3.577	<0.001
R^2^		0.036			0.045			0.035	

### Relationship Between Gender, Residence, and Information Related to COVID-19

Differences in knowledge related to educational level, gender, residence, income, and age of the respondents were investigated. The statistical outcomes revealed that all variables were non-significant in respect to age and income of the sampled population. Preventive measures to be adopted at *t*_1_ were only significant with respect to gender and residence and at *t*_2_ with respect to education (Table 6).

**Table 6:** Relationship between gender, residence and information related to COVID-19 (*n* = 384).

**Variables**	**Response**	**Percentages**	**χ^2^**	***p*-value**	**Percentages**	**χ^2^**	***p*-value**
**Gender**							
**Coronavirus general awareness at** ***t***_**1**_					**Coronavirus general awareness at** ***t***_**2**_		
Male	Having knowledge	34.4%	0.00	0.97	43.5%	0.00	0.98
	Not having knowledge	65.6%			56.5%		
Female	Having knowledge	34.2%			43.4%		
	Not having knowledge	65.8%			56.6%		
**Symptoms of COVID-19 at** ***t***_**1**_					**Symptoms of COVID-19 at** ***t***_**2**_		
Male	Having knowledge	34.4%	0.00	0.97	43.5%	3.47	0.06
	Not having knowledge	65.6%			56.5%		
Female	Having knowledge	34.2%			43.4%		
	Not having knowledge	65.8%			56.6%		
**Preventive measures to be adopted at** ***t***_**1**_					**Preventive measures to be adopted at** ***t***_**2**_		
Male	Having knowledge	27.6%	4.90	0.04^*^	53.2%	0.25	0.61
	Not having knowledge	72.4%			46.8%		
Female	Having knowledge	39.5%			50.0%		
	Not having knowledge	60.5%			50.0%		
**Residence**							
**Coronavirus general awareness at** ***t***_**1**_					**Coronavirus general awareness at** ***t***_**2**_		
Urban	Having knowledge	37.0%	1.27	0.25	46.0%	1.07	0.30
	Not having knowledge	63.0%			54.0%		
Rural	Having knowledge	31.5%			40.8%		
	Not having knowledge	68.5%			59.2%		
**Symptoms of COVID-19 at** ***t***_**1**_					**Symptoms of COVID-19 at** ***t***_**2**_		
Urban	Having knowledge	32.0%	1.33	0.24	63.0%	3.34	0.06
	Not having knowledge	68.0%			37.0%		
Rural	Having knowledge	26.6%			53.8%		
	Not having knowledge	73.4%			46.2%		
**Preventive measures to be adopted at** ***t***_**1**_					**Preventive measures to be adopted at** ***t***_**2**_		
Urban	Having knowledge	35.0%	5.07	0.02^*^	54.5%	0.60	0.43
	Not having knowledge	65.0%			45.5%		
Rural	Having knowledge	24.5%			50.5%		
	Not having knowledge	75.5%			49.5%		
**Education**							
**Preventive measures to be adopted at** ***t***_**1**_					**Preventive measures to be adopted at** ***t***_**2**_		
<11 years	Having knowledge	26.0%	1.91	0.75	36.5%	15.5	<0.01^*^
	Not having knowledge	74.0%			63.5%		
11-12 years	Having knowledge	33.1%			61.4%		
	Not having knowledge	66.9%			38.6%		
13-14 years	Having knowledge	31.3%			54.2%		
	Not having knowledge	68.8%			45.8%		
15-16 years	Having knowledge	25.0%			50.0%		
	Not having knowledge	75.0%			50.0%		
>16 years	Having knowledge	26.3%			63.2%		
	Not having knowledge	73.7%			36.8%		

* *Indicates level of significance at 0.05*.

## Discussion

The COVID-19 pandemic is one of the most challenging threats to society and public health since World War II, due to its global spread and its effects on almost every aspect of life. The media as social organization may play a vital role because it endorses adaptive measures to promote awareness and knowledge about health-related issues and encourages compliance with precautionary actions (8). The media enjoy widespread rapid access, and, therefore, serves as the major source of information for the general public during the infodemic of COVID-19. An infodemic refers to a rapid and far reaching spread of both accurate and inaccurate information. In this scenario, a global epidemic of misinformation creates severe consequences for public health. Defective and fabricated information could create panic among the masses and affect the psychological well-being of society. Hence, WHO emphasized the role of media to curb the false information and provide accurate information to people so they are well informed to act appropriately (25).

The COVID-19 epidemic in Wuhan, China, was reported worldwide, including in Pakistan (5, 13). More focused reporting was observed in the media after their global spread, as emphasized by social responsibility theory. The theory of social responsibility states that it is the professional obligation of the media to recognize the needs of the community (26). Pakistani print, electronic, and social media placed more emphasis on the adoption of preventive measures after the first case was reported in Karachi, Sindh, on 26 February 2020. The use of mass media during the initial phases of the event as a quick, effective, and evident mediator was also suggested by Rogers (27). Media outlets in Pakistan are covering the daily COVID-19 statistics. Lockdown in Pakistan led the general public to be concerned about the 2019 Novel Coronavirus (23, 24). The present research investigation evaluated the role of media awareness campaigns in shaping the perceptions of the general population toward COVID-19 in Rawalpindi. The general population utilized different types of media during the COVID-19 pandemic to access information (28). Our investigation shows that the number of users of social and electronic media increased during the coronavirus pandemic, a finding that is reinforced by further international surveys (29, 30). The number of users of print media decreased in the present study between *t*_1_ and *t*_2_. This decrease might be due to fear among users regarding COVID-19 transmission through the newspaper or by the vendor (31). Similar results were reported in India (32).

Pakistan is a male-dominated society; therefore, the majority of participants visiting the research investigation site were male (33), because tasks outside the home are considered to be the responsibility of males. Although gender inequalities have been reported in the education system in Pakistan (34), no significant differences in gender responses were observed in relation to general awareness or knowledge of the symptoms of COVID-19 in either the pre- or the post-response. Education plays an important role in understanding the medical awareness (35). The finding of low level of information in the present study in any section of the final response may be correlated with the low proportion of high educated respondents as well as the large number of respondents from rural areas. Globally, a growing body of literature reported that there was better health awareness with higher education and urban background (36, 37). Information inequalities may be linked with socioeconomic disparities because almost 90% of the respondents' families were earning <75,000 Pakistani rupees, which is an aspect closely linked with low information levels. An investigation in the United States also showed that low health awareness levels were associated with low socioeconomic status (38).

In Pakistan, many people, especially from rural areas, have the belief that there is no coronavirus and the news items related to COVID-19 are just exaggerations by the media (39). Nearly half of the sampled population in this investigation was from a rural background. The knowledge level was lower in the rural sample than in the urban sample. Although there was an increase in COVID-related knowledge among the rural population after the media awareness campaign in the country, still half of the rural group was ignorant of important aspects. The lack of awareness and misconceptions associated with COVID-19 in the rural populace may be interconnected with low literacy and the prevalence of conspiracy theories (40). Less educated individuals are more likely to believe in false myths (41). The conspiracy theories against COVID-19 are prevalent not only in Pakistan but also in other countries at a global level due to the novelty of the virus (42). Therefore, there is an urgent need that social media and other media networks are engaged in providing accurate information to people so they can act appropriately to save themselves and their next ones from COVID-19.

Overall, the respondents' knowledge related to the coronavirus increased. However, one needs to critically judge whether the anticipated outcomes were achieved solely through effective communication strategies based on the knowledge gap hypothesis (20). Moderate COVID-19 awareness among the general population has been reported in India (43). The level of awareness has been assessed as high among residents of China (44). Our study provides some hints that electronic media in particular may lead to knowledge gains as these are the most commonly used source of information not only for educated and urban people but also for people living in rural areas and with low education (45). However, the ubiquitous presence of COVID-19 in the media makes comparisons between low and high levels of exposure to media campaigns quite challenging. Furthermore, the diffusion of innovation theory also proposes that acceptance takes time and that individual's pass through various phases in the adoption procedure and may acclimatize to the concept during the later phases. Therefore, future investigations may discover improved health awareness among participants related to further items, whereas only limited progress was seen in our study, such as that related to the complications of COVID-19 (26).

The media as a modification agent can affect the behavior of individuals to enable improved well-being by acclimatizing them to the precautionary measures that halt the spread of the virus. Prevention is the essence of public health (46). China successfully controlled the epidemic in Wuhan by applying the preventive approach (47). It is the responsibility of the media to provide timely and correct information for health education and the promotion of prevention strategies. The government, in collaboration with the media, has to address the challenge of information inequalities. Rich clients of the media in Pakistan have access to high-quality and timely information. But information regarding COVID-19 is also the right of people living in rural areas and of vulnerable populations, such as refugees (48). There is a need for guidance to recognize the importance of the media for disseminating information related to the coronavirus. Health journalism requires sound knowledge related to infectious diseases. Lack of knowledge makes it challenging for journalists to describe this public health pandemic.

### Limitations

Our study sheds some light on the importance of the media in these times of the coronavirus pandemic. The results are valuable due to the large sample size. The response rate of 100%—without any missing items—indicates that the public is highly aware of the topic. However, the results need to be interpreted with caution because this research does not allow for a classical randomized or experimental study design. We were only able to distinguish between the frequency of use of various media channels. Because of the almost ubiquitous prevalence of information related to COVID-19, one might expect that even a relatively low frequency of media exposure provides information to the public. Furthermore, the results of the linear regression models indicate that there are more variables that were not included (such as health status or interest in health-related issues, and other sources of information like friends, family, healthcare providers etc.), which may further impact upon awareness and knowledge related to COVID-19.

## Conclusions

The results of this study show an overall positive effect in knowledge gains related to COVID-19 as the people acknowledged that they went to media sites for health awareness and their knowledge increased over the 4 weeks' time period. This knowledge gain ultimately encourages the use of healthy behaviors and avoids undesirable deviations in behavior among targeted populations. The investigation also highlighted the choice of media used by the participants. The numbers of social and electronic media users increased significantly during the coronavirus pandemic. It is important to communicate preventive information via the most frequently searched media to enable rapid circulation. Low preventive health awareness was associated with socioeconomically deprived groups. There is a need to develop user-friendly and indigenous communication strategies to improve the knowledge of COVID-19 among masses. Active collaboration between the government and media stakeholders is vital to safeguard the population during the COVID-19 pandemic.

The survey suggested a need for pilot studies utilizing the media during pandemics and epidemics by healthcare stakeholders for the development of rapid and timely information communication strategies. Infodemics related to infectious diseases should be addressed through effective policymaking and implementation. There is a need for inclusion of accurate information on infectious disease reporting based on rational health communication so that infodemics can be avoided in future outbreaks. Governments should address challenges to overcome health communication barriers among different social classes.

## Data Availability Statement

The raw data supporting the conclusions of this article will be made available by the authors, without undue reservation.

## Ethics Statement

The studies involving human participants were reviewed and approved by Institutional Ethical Review Board, University of the Punjab (143/IERB/PU). The patients/participants provided their written informed consent to participate in this study.

## Author Contributions

AR and RZ: conceptualized the study. AR and UH: contributed to data collection. RZ, MZ, KJW, and FF: supported in data analysis. RZ, MZ, and FF: supervised the work and supported in data analysis. AR, RZ, and UH: drafted the manuscript. All authors contributed to revising the manuscript and approved the final manuscript.

## Funding

We acknowledge support for the publication costs by the Open Access Publication Fund of Bielefeld University.

## Conflict of Interest

The authors declare that the research was conducted in the absence of any commercial or financial relationships that could be construed as a potential conflict of interest.

## Publisher's Note

All claims expressed in this article are solely those of the authors and do not necessarily represent those of their affiliated organizations, or those of the publisher, the editors and the reviewers. Any product that may be evaluated in this article, or claim that may be made by its manufacturer, is not guaranteed or endorsed by the publisher.
