# Supplementary material for: Role of the Media in Health-Related Awareness Campaigns on Perception of COVID-19: A Pre-post Study in the General Population of Pakistan
Source: Front Public Health. 2021 Nov 12;9:779090. doi: 10.3389/fpubh.2021.779090 (PMC8632810; doi:10.3389/fpubh.2021.779090)
Supplement: Supplementary file 1 [file Data_Sheet_1.PDF]

Date of response……………………….

Questionnaire code……………………

COVID-19 Awareness Questionnaire

Thank you very much for your willingness to participate in this study. Your opinion will be helpful in assessing the Corona (COVID-19) pandemic awareness at national level. You may know that the corona epidemic in China resulted in sickness and deaths among the general population. This questionnaire will assess your media use frequency for health awareness and knowledge related to COVID-19. You will be contacted again for another response through your registration record in future. I assure you that your responses are completely anonymous.

Kindly encircle your response.

Sociodemographics

Age (in years) ………………………………

Gender

a) Male b) Female

Marital status

a) Married b) Unmarried c) Widowed/divorced

Residence

a) Urban b) Rural

Education

a) <10 years of education b) 11–12 years of education c) 13–14 years of education

d) 15–16 years of education e) ≥17 years of education

Monthly income (in PKR)

a) <25,000 b) 25,000–50,000 c) 50,001–75,000 d) 75,001–100,000 e) >100,000

Media use for health awareness

How frequently have you used the following media sources for health awareness in the past 3 months?

Social media (Facebook, Whatsapp, Instagram, Tiktok, any other) use

a) Daily b) Weekly c) Not follow

Electronic media use (television, radio, any other) use

a) Daily b) Weekly c) Not follow

Print media (newspaper, magazine, any other) use

a) Daily b) Weekly c) Not follow

COVID-19 awareness

1. Do you believe coronavirus is a contagious viral disease?

a) Yes b) No

2. Do you believe coronavirus spreads via droplet infection?

a) Yes b) No

3. Do you believe that coronavirus spreads through coughing and sneezing of the infected person?

a) Yes b) No

4. Do you believe that coronavirus treatment options are only supportive?

a) Yes b) No

5. Do you know about vaccine available against coronavirus virus?

a) Yes b) No

COVID-19 symptoms and complications

6. Do you know about fever as a symptom of COVID-19?

a) Yes b) No

7. Do you know about cough as a symptom of COVID-19?

a) Yes b) No

8. Do you know about body aches as a symptom of COVID-19?

a) Yes b) No

9. Do you know about shortness of breath as a symptom of COVID-19?

a) Yes b) No

10. Do you know about pneumonia as a complication of COVID-19?

a) Yes b) No

11. Do you know about organ failure as a complication of COVID-19?

a) Yes b) No

Preventive measures for COVID 19

12. Do you think that frequent hand-washing with soap for 20 seconds is necessary to prevent COVID-19?

a) Yes b) No

13. Do you think that following cough/sneeze etiquette (cover your mouth and nose with a tissue when coughing or sneezing, dispose of the tissue after use, perform hand hygiene) is necessary to prevent COVID-19?

a) Yes b) No

14. Do you think avoiding social contact with sick people is necessary to prevent COVID-19?

a) Yes b) No

15. Do you think use of face mask is necessary to prevent COVID-19?

a) Yes b) No

16. Do you think use of sanitizer is necessary to prevent COVID-19?

a) Yes b) No

17. Do you think isolation of suspected cases is necessary to prevent COVID-19?

a) Yes b) No

Lockdown effect

18. Do you believe lockdown in countries during COVID-19 enables people to follow social distancing?

a) Yes b) No

19. Do you believe that lockdown in countries during COVID-19 results in more self-protection?

a) Yes b) No

Any comments

……………………………………………………………………………………………………

……………………………………………………………………………………………………

……………………………………………………………………………………………………

……………………………………………………………………………………………………

……………………………………………………………………………………………………

……………………………………………………………………………………………………
